# Perceptions of women’s HIV risk and partner HIV risk behaviors in substance using women with criminal justice involvement

**DOI:** 10.15761/CBHC.1000109

**Published:** 2015-12-21

**Authors:** Doreen Domenica Salina, Daphna Ram, Leonard A. Jason

**Affiliations:** 1 Department of Psychiatry and Behavior, Northwestern University, Chicago, IL, USA; 2 Center for Community Research, DePaul University, Chicago, IL, USA

**Keywords:** HIV/AIDS, incarcerated women, HIV risk behaviors, substance abuse disorders

## Abstract

This article explores the perceptions of STI/HIV risk based on engagement in risk behaviors in a sample of women with substance use disorders and criminal justice involvement. We examined variables associated with higher risk of contracting STI/HIV: having more than one current sex partner, injecting drugs, and trading sex. We also examined risk variables associated with intimate relationships: whether a partner had ever been in prison, injected drugs, or shared needles. Findings reveal that certain high-risk behaviors influenced participant perceptions of HIV risk: having more than one current sex partner, having a partner who injected drugs, having a partner who had sex with a man, or having a partner who had been tested for HIV. Participants who were uncertain about whether a partner had engaged in risk behaviors had significantly higher worry and perceptions of HIV risk than participants who were certain of partners’ risk behaviors. The implications of these findings for tailoring effective interventions for high-risk women are addressed.

HIV/AIDS disproportionately impacts women with substance use disorders who are justice-involved (e.g., arrested, incarcerated, paroled or placed on probation). In order to effectively reduce high numbers of infections, health interventionists need to understand what beliefs and behaviors contribute to these high rates of this preventable chronic illness. The goal of the present study is to examine HIV risk behaviors in one group of women with characteristics associated with increased HIV risk: those with substance use disorders [1,2] within communities of color [3], having high rates of sexual abuse [4], and histories of incarceration [5]. In addition, this study will assess women’s own perception of their HIV risk relative to other women and how they perceive the effect of the specific risk behaviors of intimate partners.

Justice-involved women have more risk factors associated with acquiring HIV, including injection drug and other drug use, commercial sex work, untreated mental illness, and lower socioeconomic status. Furthermore, it is well known that women in the criminal justice system suffer from multiple health risk factors such as poverty and lack of stable housing, and that many are victims of childhood and adult physical and/or sexual trauma [6,7]. In general, women with substance use disorders have higher levels of general health risks for many problems including HIV/AIDS [8].

The intimate relationships of women who have been involved with the criminal justice system relationships have characteristics that place women especially high risk for contracting STIs, including HIV/AIDS. Reliance on male intimate partners for access to survival needs may set a significant power differential which decreases the woman’s perceived ability to negotiate risky situations and behaviors. Indeed, women with substance use disorders have been found to experience higher rates of domestic violence associated with attempting to negotiate condom use [9], and these instances of physical violence from a male intimate partner are associated with a lack of consistent condom use [10,11]. Fear of physical and financial retribution, compounded by the unequal power balance inherent in these relationships, may prevent women from behaving assertively and insisting on condom use [12], leading to HIV/STI risk.

Yet consistent condom use may stem from a woman’s lack of awareness regarding her partner’s HIV risk behaviors [13–15]. However, there is also evidence that even having knowledge of a partner’s HIV risk behaviors may not be sufficient to encourage consistent condom use [16]. Therefore, it is possible that there are other factors influencing the relationship between a woman’s knowledge of a partner’s HIV risk behaviors and using condoms; specifically, a woman’s perception of her own HIV risk in relation to her perception of her partner’s risk behaviors.

The relationship between the aforementioned characteristics of women (i.e., those within communities of color, those with high rates of sexual abuse, those with substance use disorders, and those with histories of incarceration) and HIV risk is intricate and complex. In order to obtain a clearer picture of the risks for this population of women, and therefore develop effective interventions to reduce HIV risk, we examine the HIV risk behaviors of women with these characteristics. In addition, we examine perceptions of intimate partners’ risk behaviors in a sample of justice-involved women with substance use disorders. The goal of the present study is to examine both the participants’ specific HIV risk behaviors, and their partners’ risk behaviors in a sample of justice-involved women with substance use disorders.

## Materials and method

### Participants

Two hundred women who reported being in recovery from a substance-use disorder and who had involvement with the criminal justice system within the preceding two years were recruited from multiple sites in metropolitan Chicago and its suburbs from 2008 to 2011. This was part of a parent study examining the role of democratically run recovery homes on preventing relapse and recidivism. Recruitment sites included Cook County Jail and multiple substance abuse treatment sites throughout Chicago, the surrounding suburbs, and Northern Illinois. These treatment sites included residential inpatient programs of varying length, outpatient treatment centers, as well as neighborhood Alcoholics Anonymous and Narcotics Anonymous meeting places. While research staff actively visited these sites to recruit participants for the study, they also posted flyers in places that might provide some form of services to substance using women who were formerly or currently justice-involved. Participants were also recruited using snowball techniques, which permitted other participants to refer women to the study. Most women who learned about the project agreed to participate in the study, with the exception of a very few women who declined. No women were refused participation by study personnel.

### Procedures

At baseline we collected general demographics on all participants, including race, education, marital status, housing and employment status in the last year, as well as income history and its source. We collected data on the types of criminal offenses for which participants had been arrested and charged, their previous criminal histories, the number of arrests, and months incarcerated. Consistent with existing literature, the 200 participants in this study were primarily from communities of color, undereducated, and under employed (see Table 1 for a complete list of demographic variables). As noted in the inclusion criteria, all participants reported current or previous criminal justice involvement. The sample consisted of mostly African American women (74.5%; n=149), and most of the sample was currently unemployed (66%; n= 132). Only 22.8% (n=45) reported receiving their primary income from legal employment; the next highest primary sources of financial support were selling drugs (17.3%; n=34) and sex work/prostitution (14.7%; n= 29). Of the women who were employed (34%; n= 68), 26.5% (n=18) reported their major source of income over the last year was a result of illegal activities, including selling drugs and prostitution/sex work.

Also at baseline, participants were asked to complete a tracking information sheet in order to be followed longitudinally and to voluntarily obtain a HIV test at a local health department clinic or private testing site. If requested, staff accompanied the participant to the clinic for the HIV test. The HIV test was usually scheduled on the same day as the baseline interview. HIV testing was not a requirement to participate in the study.

Participants received $45 in grocery store gift cards for participating in the initial interviews. This study was reviewed and approved by the study institution’s IRB board and followed the provisions of the Federal regulations in Subpart C of 45 CFR 46. The IRB approved the amount of compensation as being consistent with the time spent completing the interview. Participants also received transportation passes to travel to the interview and an additional transportation pass if they chose to receive HIV testing.

### Measures

Each participant was individually interviewed by a trained research assistant in a private space. Data were collected through participants’ self- reports regarding their behaviors related to HIV risk. We also asked about their perceptions of their primary partners’ monogamy and sexual behaviors outside of the intimate relationship. Other relevant variables included the number of lifetime sexual partners, histories of sexually transmitted infections (STI), condom frequency use, and the self-report of forced or coerced sexual behavior. Specifically, we asked participants about sexual victimization and the age at which it occurred and the frequency of these incidents. We also queried whether participants had ever traded sex for money or drugs and how frequently they had traded in the past.

### Risk behavior survey

This scale obtains self- reported information regarding sexual and drug use histories. The Risk Behavior Survey (RBS) takes 30 to 45 minutes to complete, and covers demographic information; drug use history; sexual behaviors; condom use; exchange of sex for drugs, money, or both; and HIV- test history [17]. Data from selected sections of this measure are included in this article.

### HIV counseling, testing and referral

HIV testing data were collected with the question, ‘Have you ever been tested for HIV?’ HIV status information was initially collected via self-report, and, additionally, all women who reported a negative or unknown serostatus were offered voluntary HIV testing and counseling, which were conducted at local health sites. HIV testing was completely voluntary and it was not required to participate in the study. All federal, state, and local statutes were strictly followed with respect to confidentiality and disclosure of test results.

### Assessment of participant’s HIV/STI risk

Participants were asked to report their own high-risk behaviors, including injecting drugs, trading sex, and whether they currently had more than one sexual partner.

### Assessment of partner risk

Participants were asked about the HIV/STI risk behaviors/characteristics of their male partner(s), such as whether they had been incarcerated, whether they had used drugs intravenously, if they had impregnated someone else during the relationship, or if the participant knew that their male partner had had sex with a man/men. Responses included “definitely yes”, “definitely no”, or “not sure”.

### Assessment of participant worry

Participants were asked how much they worried about getting HIV from their partners (“How much do you worry about getting HIV from you partner?”). Responses ranged from 0 (“not at all”) to 3 (“quite a lot”). Women were asked how much they worried about contracting HIV compared to their other problems (“Compared to other problems in your life, how much do you worry about contracting HIV?”). Responses ranged from 0 (“a lot less”) to 4 (“a lot more”). Finally, participants were asked about how they compared their HIV risk to other women (“Compared to other women in Chicago, how would you rate your risk of getting HIV?”). Responses ranged from 0 (“much lower risk”) to 4 (“much higher risk”).

## Results

### Participant HIV risk behaviors and knowledge about partner risk

We examined the prevalence of engaging in HIV risk behaviors in our sample, such as trading sex and injecting drugs. The majority of our participants had traded sex (67.7%; n=128), and a substantial number had injected drugs (30.5%; n=61). We also examined participant knowledge of partner HIV risk behaviors. The majority of women in our sample (75.5%; n=148) knew that their partner had had sex with someone else while in a committed relationship, and 42.6% (n=83) knew that their partner had given them a sexually transmitted disease. A complete list of the prevalence of HIV risk behaviors in our sample, and of knowledge of partner risk, is provided in Table 2.

### HIV worry and perception of risk

In general, women in the sample had low worry regarding contracting HIV from a partner (M=.85; SD=1.13), low worry regarding contracting HIV compared to other problems in their life (M= .82; SD=1.26), and rated their risk of contracting HIV as low compared to other women in Chicago (M=.84, SD= 1.12).

In order to explore whether participant risk behaviors and knowledge of partners’ high-risk HIV behaviors influenced participants’ worry and perception of risk regarding contracting HIV, we ran a multivariate ANOVA (MANOVA) with the participant risk and partner risk variables predicting the three questions about HIV worry and risk perception (i.e., “How much do you worry about getting HIV from your partner?”; “Compared to other women in Chicago, how would you rate your risk of getting HIV?”; “Compared to other problems in your life, how much do you worry about getting HIV?”). The complete list of the risk behaviors used in the MANOVA is included in table 2.

Given the exploratory nature of this study, we then employed backward elimination to comprise a smaller set of variables that best predict participant worry and perception of risk of HIV. Four variables predicted participant worry and perception of risk for contracting HIV: whether or not the participant currently had more than one sex partner, knowing whether a participant’s partner had had sex with a man, knowing whether a participant’s partner had used IV drugs, and knowing whether a participant’s partner had been tested for HIV.

**Figure 1:**
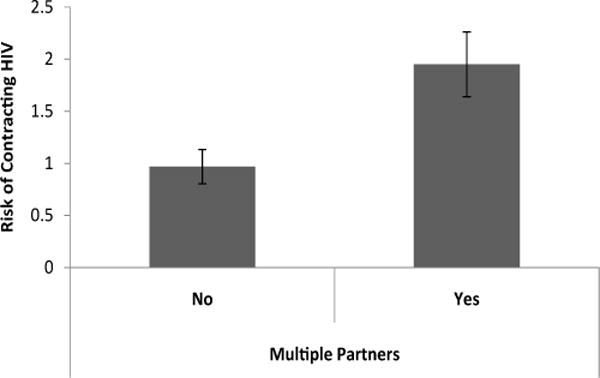
Perceived risk of HIV contraction compared to other women in Chicago based on having multiple sex partners.

**Figure 2:**
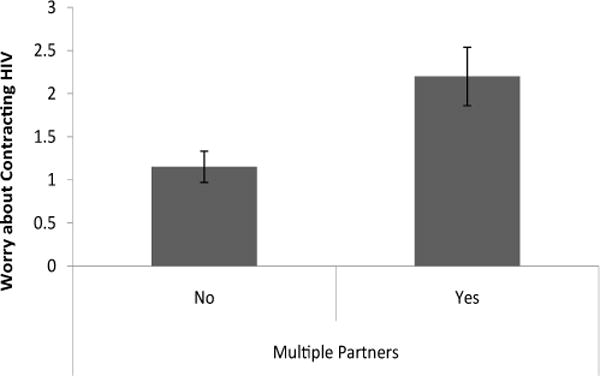
Level of worry about contracting HIV in comparison to other problems based on having multiple sex partners.

Currently having more than one sexual partner had a significant effect on worry about HIV and perception of HIV risk [Λ=.92, F (3, 171) = 5.17, p < .05]. Follow-up univariate tests revealed that women who did not have more than one current sexual partner rated their risk of getting HIV significantly lower (M=.97, S.E. = .17) than other woman in Chicago [t(173) =3.49, p < .01, d = .89] compared to women who had more than one current sexual partner (M=1.95, S.E.=.31; Figure 1). Women who currently had more than one sexual partner also had higher worry about getting HIV (M=2.20, S.E. = .34) compared to other problems in their life [t(173) = 3.41, p < .01, d = .87] than women who did not have more than one sexual partner (M=1.15, d=.18; Figure 2).

**Figure 3:**
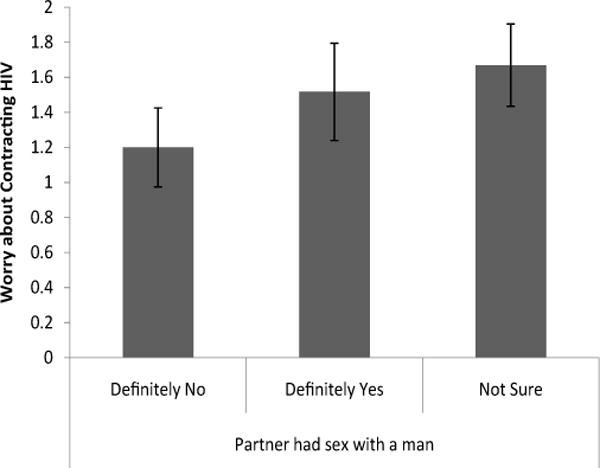
Level of worry about contracting HIV from a partner based on knowledge of whether partner had sex with a man.

Knowing whether a partner had had sex with a man had a significant effect on worry about HIV and perception of HIV risk [Λ=.92, F(6, 344) = 3.05, p < .01]. Follow-up univariate tests showed that women who were not sure if a partner had sex with a man reported being more worried [t(173)=2.36 p < .05, d = .41] about getting HIV from a partner (M=1.67, S.E.= .24) than women who knew that a partner did not have sex with a man (M = 1.20, S.E. = .23; Figure 3).

**Figure 4:**
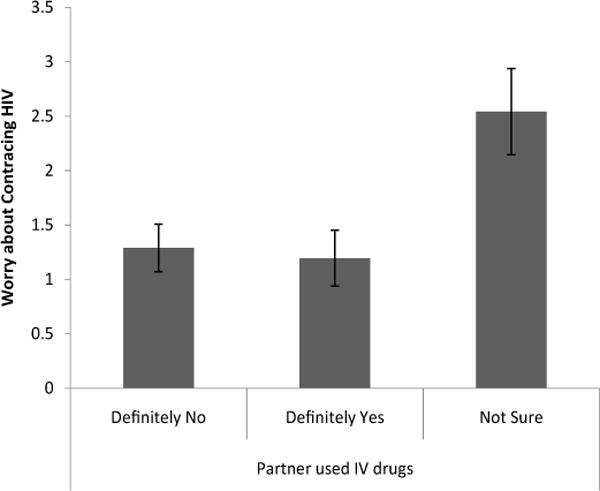
Level of worry about contracting HIV in comparison to other problems based on knowledge of whether a partner used IV drugs.

Knowing whether a partner had used IV drugs had a significant effect on worry about HIV risk [Λ=.88, F(6, 344)=3.80, p<.01]. Follow-up univariate tests revealed that women who were not sure if a partner had used IV drugs reported significantly more worry (M = 2.54, S.E=.40) about getting HIV compared to other problems in their life [t(173)=3.26, p < .01, d = 1.07] than women who knew that a partner had used IV drugs (M=.1.20, S.E.=.26) and significantly more worry about getting HIV compared to other problems in their life [t(173) = 3.30, p < .01, d = 1.00] than women who knew that a partner had not used IV drugs (M=.1.29, S.E=.22; Figure 4).

**Figure 5:**
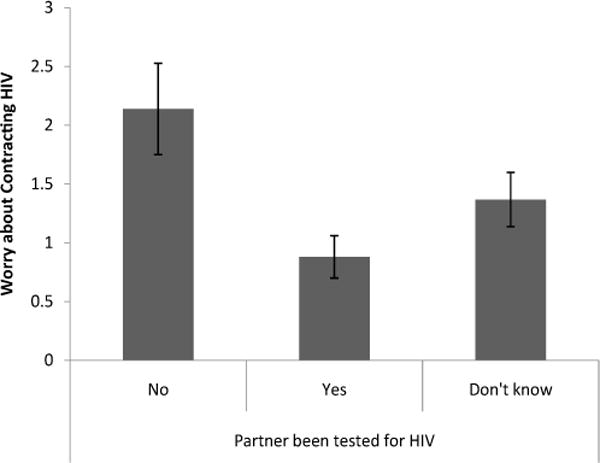
Level of worry about contracting HIV from a partner based on knowledge of whether partner has been tested for HIV.

**Figure 6:**
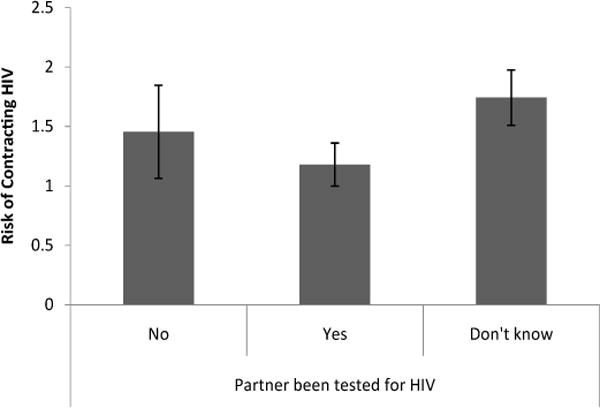
Perceived risk of HIV contraction based on knowledge of whether a partner had been tested for HIV.

**Figure 7:**
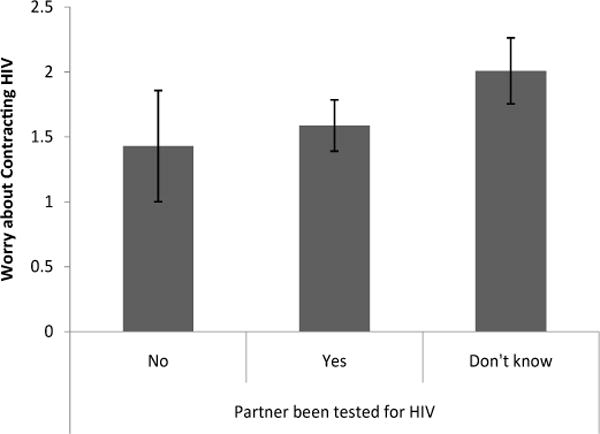
Level of worry about contracting HIV in comparison to other problems based on knowledge of whether a partner had been tested for HIV.

Knowing whether a partner had been tested for HIV had a significant effect on worry about HIV and perception of HIV risk [Λ=.86, F(6, 344)=4.17, p < .01]. Women who knew that a partner had been tested for HIV were significantly less worried [t(173)=3.35, p <.01, d = 1.16] about getting HIV from a partner (M= .88, S.E. = .18) than women who knew that a partner had not been tested for HIV (M=2.14, S.E=.39) and significantly less worried [t(173) = 2.50, p < .05, d =.44] about getting HIV from a partner than women who didn’t know whether a partner had been tested for HIV (M=1.37, S.E.=.23) (Figure 5). Women who knew a partner had been tested for HIV rated their risk of getting HIV compared to other women in Chicago (M=1.18, S.E.=.18) significantly lower [t(173)=2.87, p<.01, d=.51] than women who weren’t sure whether a partner had been tested for HIV (M=1.74, S.E.=.23) (Figure 6). Finally, women who knew that a partner had been tested for HIV rated their worry about getting HIV compared to other problems in their life (M=1.59, S.E.=.20) marginally lower [t(173)=1.97, p =.51, d =.35] than woman who did not know whether a partner had been tested for HIV (M = 2.01, S.E. = .25) (Figure 7).

## Discussion

This study supports previous work showing that justice-involved women engage in a variety of HIV risk behaviors, such as trading sex as a survival mechanism or to support their drug use, and injecting drugs. The women in our study also had had relationships with partners who had engaged in high-risk sex and drug use behaviors, such as sharing needles and having sex outside a committed relationship. However, more understanding is needed as to how justice-involved women’s knowledge of their male partners STI/HIV risk behaviors and their own perception of their personal risk contribute to actual risk.

One of the more interesting findings was that the majority of the women in our sample did not believe they were at high risk of contracting HIV and did not worry about contracting HIV. Overall scores regarding worry and risk of contracting HIV were low, despite the consistently high levels of engagement of risk behaviors by themselves and their intimate partners. Furthermore, engaging in high risk behaviors, either directly themselves or indirectly through high-risk sexual partners, did not substantially influence women’s perception of their HIV risk or worry about contracting HIV. Of the 13 variables we examined, only four were found to be related to HIV contraction risk and worry: whether the participant had more than one sexual partner currently, knowing whether a partner had been tested for HIV, knowing whether a partner had had sex with a man, and knowing whether a partner had used IV drugs.

In addition to being in relationships with high-risk partners, a substantial number of women were unsure whether previous partners had engaged in certain risk behaviors. These findings suggest a lack of communication between these women and their partners, which is problematic, as knowing a partner’s HIV risk behaviors is critical in reducing and preventing HIV risk. Furthermore, in our sample, an individual’s feelings of certainty regarding a partner’s risk behavior played a strong role in a woman’s perception of her HIV risk. Generally, uncertainty regarding whether a partner had engaged in specific HIV risk behaviors was associated with higher concern about contracting HIV compared to other problems and feeling a higher risk of contracting HIV compared to other women in Chicago. These findings suggest that knowledge of a partner’s high-risk behaviors in one domain may mean knowledge regarding other domains as well, lending support for the notion that that communication within relationships is of key importance in an individual’s assessment of his or her risk.

Overall, the lack of correspondence between a women’s engagement in risk behaviors, either directly or indirectly through high-risk sexual partners, and the perception of her HIV risk has important implications for interventions and policy. Whereas previous work suggests that fear of intimate partner violence or abandonment [18] may contribute to high-risk HIV behaviors such as low condom use, our work provides preliminary evidence that women may not necessarily be accurately processing the extent to which partner risk may influence their own level of risk. The four variables that significantly influenced perception of partner risk represent some of the most direct routes of HIV transmission, suggesting that the women are somewhat accurately considering the implications of partner risk; however, the overall low level of worry regarding HIV contraction in this high-risk sample still suggests a disconnect between a woman’s behavior and perceived risk. Intervention programs should place a stronger emphasis not only on the fact that knowing about a partner’s risk behaviors is important, but that also understanding the link between a partner’s risk and one’s own risk is imperative.

Our findings also suggest that promoting communication within intimate relationships is an important part of HIV intervention and prevention programs, as many women stated that they were unsure whether a partner engaged in certain high-risk behaviors. This may be due to the fact that compared to men, women are often less powerful in their interpersonal relationships and may fear loss of essential resources, violence, or other types of retaliation if they question their partners about their risk behaviors. Previous work has found that substance-using women who endorsed traditional gender roles or who were in relationships with dominant, controlling men, reported feeling less power within the relationships [19]. Ideally, prevention programs should address the significant inequity of power between the genders that contribute to women staying involved with intimate partners who are destructive to them through external relationships, chronic drug use, unprotected sex, and interpersonal violence.

Women who have participated in an HIV/STI prevention program typically know a great amount about STI/HIV transmission. Our findings that women rated their overall risk of contracting HIV as low even though they were exposed to high-risk behaviors may be due to the fact that our participants were asked retrospectively about their partner’s risk behaviors and about their own current assessment of risk. Furthermore, participants were interviewed right after they had left substance abuse treatment, which may have led them to be overly optimistic in their assessments of their own risk.

It is important to note that there are cultural specific considerations when developing strategies designed to empower women to exercise better care of themselves. Because, one shoe does not fit all, there are a number of potential barriers to implementing or delivering the types of interventions suggested. For example, certain cultures might not encourage their members to seek help from sources outside the family, or they might be suspicious of outside interventions. These and other cultural practices need to be considered when designing interventions.

In general, HIV testing programs in the Chicago area have been successful as a large percentage of women in this sample had been tested for HIV and received results. However, very few HIV prevention and intervention programs evaluate whether women are able to understand the imminent risk to themselves. Furthermore, few programs promote skills such as assertiveness and self-efficacy in women. Interventions need to explicitly promote the message that women do not have to be or remain victims and that expecting one’s intimate partner to practice monogamy is not an unreasonable demand. These skills, while useful for HIV prevention efforts, are also more specific to reducing many of the challenges and social ills that affect this population, including substance abuse, community violence, and repeat sexual victimization. Without the perception that each woman can actually and effectively control her level of STI/HIV risk, prevention programs that address these complex problems in a simple, manualized fashion are likely to be less effective when women are actually in a situation that requires complex intrapersonal skills. Interventions need to include an understanding that for many women, behavioral skills alone will not be sufficient to reduce their risk of STI/HIV transmission. This is especially true for marginalized women who suffer from substance use disorders and repeated involvement in the criminal justice system. These women, who often demonstrate tremendous resilience in the face of overwhelming circumstances, require a broader focus than the provision of behavioral skills. Interventions for this group should include opportunities for improving self-efficacy and access to tangible resources. The cognitive framing of the intervention messages can be modified to still promote focus on the behavioral aspects of HIV risk reduction strategies, but they must include an understanding why so many of these strategies are perceived by justice-involved women as difficult.

## Figures and Tables

**Table 1:** Socio-demographic Variables of Participants at Baseline.

Variables	Total (*n*=200)
**Age, mean (SD), yrs**	39.94 (8.58)
**Race/Ethnicity, % (No.)**	
Black/African American	74.5 (149)
White/Caucasian	22.5 (45)
Hispanic/Latina	2.0 (4)
Other	1.0 (2)
**Education, % (No.)**	% *(n)*
8^th^ grade	3.0 (6)
9^th^ grade	4.5 (9)
10^th^ grade	11.0 (22)
11^th^ grade	22.0 (44)
12^th^ grade	18.0 (36)
GED	9.5 (19)
Some college	23.0 (46)
Vocational	4.5 (9)
College	3.5 (7)
Postgraduate	1.0 (2)
**Employment, % (No.)**	
Unemployed	66.0 (132)
Employed	34.0 (68)
**Primary sources of income, % (No.)**	
Job/employment	22.8 (45)
Selling drugs	17.3 (34)
Selling or trading sex (prostitution)	14.7 (29)
Other Illegal Activities	11.2 (22)
Family	7.6 (15)
Disability	6.6 (13)
Welfare or Public Assistance	6.6 (13)
Ex-partner/ex-spouse	5.6 (11)
Current partner/sexual partner	5.6 (11)
Unemployment Compensation	1.5 (3)
Other	0.5 (1)

**Table 2:** Risk Factors of Participants and their Partners.

Risk Factors of Participants, % (No.)	Yes		No	
Have you ever injected?	30.5 (61)		69.5 (139)	
Are you currently involved in a sexual relationship with more than one partner?	9.0 (17)		91.0 (171)	
Have you ever traded sex for drugs or money?	67.7 (128)		32.3 (61)	
**Risk Factors of Partners, % (No.)**	**Definitely Yes**	**Definitely No**		**Not Sure**
Had sex with someone else while you were in a committed relationship?	75.5 (148)	13.3 (26)		11.2 (22)
Had unprotected sex with someone else while you were in a committed relationship?	60.0 (117)	16.9 (33)		23.1 (45)
Got someone else pregnant while you were supposed to be in a committed relationship?	23.4 (45)	57.8 (111)		18.8 (36)
Had sex with a man (either publicly admitting or on the “down low”)?	14.8 (29)	59.7 (117)		25.5 (50)
Ever served a prison sentence?	69.4 (136)	27.0 (53)		3.6 (7)
Served a prison sentence in the past 5 years?	49.5 (96)	46.4 (90)		4.1 (8)
Given you a sexually transmitted disease like gonorrhea, syphilis, the clap, Chlamydia, Trichomonas?	42.6 (83)	56.4 (110)		1.0 (2)
Been diagnosed with Hepatitis C?	9.2 (18)	79.0 (154)		11.8 (23)
Used IV Drugs?	24.0 (47)	69.4 (136)		6.6 (13)
Shared needles while using IV drugs?	16.2 (31)	73.8 (141)		9.9 (19)
Has your partner ever been tested for HIV?	72.2 (135)	4.8 (9)		23.0 (43)
